# Cortical bone mechanics technology signal quality maintains robustness across a range of biometric profiles

**DOI:** 10.1093/jbmrpl/ziaf116

**Published:** 2025-07-09

**Authors:** Andrew Dick, Max Stoeckel, Massimo Ruzzenne, Tony von Sadovszky, Janet E Simon, Leatha A Clark, Stuart J Warden, Todd M Manini, Charalampos Lyssikatos, Tiffani Hart, Brian C Clark

**Affiliations:** OsteoDx Inc., Athens, OH 45701, United States; OsteoDx Inc., Athens, OH 45701, United States; Department of Mechanical Engineering, University of Colorado, Boulder, CO 80309, United States; TechGrowth Ohio, Athens, OH 45701, United States; Ohio Musculoskeletal and Neurological Institute (OMNI), Ohio University, Athens, OH 45701, United States; Department of Athletic Training, Ohio University, Athens, OH 45701, United States; Ohio Musculoskeletal and Neurological Institute (OMNI), Ohio University, Athens, OH 45701, United States; Department of Biomedical Sciences, Ohio University Heritage College of Osteopathic Medicine, Athens, OH 45701, United States; Department of Physical Therapy, School of Health and Human Sciences, Indiana University Indianapolis, Indianapolis, IN 46202, United States; Indiana Center for Musculoskeletal Health, School of Medicine, Indiana University Indianapolis, Indianapolis, IN 46202, United States; Institute on Aging, University of Florida, Gainesville, FL 32610, United States; Indiana Center for Musculoskeletal Health, School of Medicine, Indiana University Indianapolis, Indianapolis, IN 46202, United States; Ohio Musculoskeletal and Neurological Institute (OMNI), Ohio University, Athens, OH 45701, United States; Ohio Musculoskeletal and Neurological Institute (OMNI), Ohio University, Athens, OH 45701, United States; Department of Biomedical Sciences, Ohio University Heritage College of Osteopathic Medicine, Athens, OH 45701, United States

**Keywords:** osteoporosis, BMD, cortical bone mechanics technology (CBMT), mechanical response tissue analysis (MRTA), bone strength, fracture

## Abstract

Current methods of diagnosing osteoporosis, such as DXA, have limitations in predicting fracture risk. Cortical bone mechanics technology (CBMT) offers a novel approach by using a three-point bend test with multifrequency vibration analysis to directly measure ulnar bending stiffness and calculate flexural rigidity, a mechanical property highly predictive of whole-bone strength under bending conditions. Cortical bone mechanics technology targets the diaphyseal ulna, a site composed primarily of cortical bone, enhancing its specificity for cortical bone quality. In this study of 388 postmenopausal women, we developed and validated a 20-point signal quality indicator (SQI) scoring system to quantify CBMT signal quality and evaluated its relationship to biometric characteristics. The SQI was developed through expert assessment of representative frequency response function (vibration data) trials and refined over 17 iterations. The final system achieved excellent classification performance (AUC = 0.974; sensitivity, specificity, and accuracy all >97%). A total of 22 740 trials were collected across 758 total arm tests, sampling 10 ulnar sites per arm under three vibration amplitudes. Two expert analysts evaluated signal features associated with high signal quality. The resulting SQI is fully automated and provides real-time feedback. All correlations between SQI scores and biometric attributes were weak or very weak (|ρ| < 0.30). The correlations with body weight (ρ = −0.11), BMI (ρ = −0.12), ulnar BMD (ρ = −0.17), CBMT-derived flexural rigidity (ρ = −0.28), and grip strength (ρ = 0.17) were statistically significant (*p* < .05) but remained small in magnitude. SQI scores were modestly lower in individuals with higher BMI or flexural rigidity (~2 to 3 points), but values remained in the acceptable-to-good range. This study introduces a robust, automated CBMT signal quality metric and demonstrates that its performance remains stable across a broad range of biometric profiles, supporting its application in both clinical and research settings.

## Introduction

Osteoporosis is characterized by low bone mass and structural deterioration which increase bone fragility and increase fracture risk, particularly in post-menopausal women and aging populations.^1^^,^^2^ The personal and economic burden of osteoporosis is staggering, with approximately 2.3 million osteoporotic fractures occurring annually in the U.S. alone,^3^ resulting in an estimated total cost of $72 billion USD in 2025 (adjusted from 2018 values for inflation).^4^

DXA is the current standard for assessing bone health, providing measures of areal BMD (aBMD). However, its ability to predict fracture risk is limited.^5–16^ For example, a large prospective study of >14 000 men and women, followed for up to 20 yr, found that the majority of fragility fractures—79% in men and 75% in women—occurred in individuals with normal or osteopenic aBMD, rather than in those classified as osteoporotic.^16^ Similarly, patients with a history of fractures remain at significantly higher risk of future fractures compared to those with similar aBMD but no prior fractures, even after adjusting for other risk factors.^17^^,^^18^ While bisphosphonate therapy improves bone strength and reduces fracture risk, <18% of the reduction in vertebral fractures can be attributed to increases in aBMD measured by DXA.^19^^,^^20^ Moreover, data from a prospective study of >7000 men and women (mean follow-up: 4.6 yr) demonstrate that FN aBMD has poor sensitivity for fracture prediction.^11^ These findings underscore the significant limitations of DXA-derived aBMD as a standalone assessment of bone health and highlight the urgent need for improved diagnostic and predictive tools to better identify individuals at risk and ensure appropriate treatment.^10^^,^^21^^,^^22^

aBMD provides information only about the quantity of mineral in bone, which represents just one aspect of bone strength.^5^^,^^23^^,^^24^ Recent advancements in bone biomechanics research have opened new methods to quickly, non-invasively, and painlessly assess the mechanical properties of human ulnar cortical bone. Specifically, cortical bone mechanics technology (CBMT) employs a three-point bend test combined with multifrequency vibration analysis to conduct dynamic, structural, and mechanical bending tests of the ulna (for reviews see^12^^,^^25^). The CBMT system analyzes the frequency response functions of the resulting vibrations, fitting them to a mathematical model of the skin-bone mechanical system. This approach enables noninvasive, direct, and functional measurements of bending stiffness, which are then used to calculate flexural rigidity (*EI*), a key mechanical property that governs a bone’s resistance to deformation under bending loads and strongly predicts its failure strength.^12^^,^^26^^,^^27^

From an engineering perspective, flexural rigidity (EI) represents a structure’s resistance to bending and is defined as the product of the material’s elastic modulus (E), reflecting its intrinsic stiffness, and the area moment of inertia (I), which captures the distribution of cross-sectional area relative to the neutral axis. Together, E and I integrate material properties and structural geometry, 2 key factors influencing flexural behavior in both engineered and biological systems.^28^^,^^29^ This quantity governs how a structure resists deformation under bending loads and is a principal determinant of whole-structure performance in flexural contexts, including long bones subjected to habitual loading.^28^^,^^30^ In prior ex vivo validation studies using cadaveric human ulnae, CBMT-derived EI demonstrated an exceptionally strong correlation with mechanical failure load (*R*^2^ = 0.99), highlighting its value as a surrogate for whole-bone strength in bending.^26^ While CBMT does not assess all aspects of fracture resistance, such as cortical fracture toughness or crack propagation behavior, it provides direct, quantitative insight into the mechanical integrity of cortical bone.

Cortical bone mechanics technology targets the mid-point of the ulna for several key reasons. First, this region is primarily composed of cortical bone, which is particularly relevant since most bone loss after age 65 occurs in the cortical compartment.^31^ Second, the ulna’s anatomy makes it well-suited for a bending test, as its proximal and distal ends can be rigidly supported.^32^ Third, fragility fractures of the wrist, including those involving the ulna, are strong predictors of future hip fractures, highlighting a correlation between wrist and hip fragility.^33–35^

Mechanical response tissue analysis (MRTA), an earlier vibration-based technology developed decades ago, demonstrated strong associations between flexural rigidity and bone structural integrity^36^^,^^37^ as well as its relevance in disease (eg, osteogenesis imperfecta^38^ and secondary hyperparathyroidism^39^), and degenerative conditions.^40^^,^^41^ However, despite its scientific promise, MRTA faced commercialization challenges that ultimately hindered its widespread adoption.^25^^,^^27^^,^^42^^,^^43^ Cortical bone mechanics technology builds upon the foundational principles of MRTA while incorporating significant technological advancements, including robotics and automated protocols, to improve probe placement precision, enhance testing efficiency, and increase patient comfort. Additionally, CBMT employs novel algorithms for real-time data analysis, expediting result delivery (for more details, see^12^^,^^27^^,^^32^).

Despite these advancements, little is known about factors influencing CBMT outcomes. In particular, the impact of biometric attributes such as BMI, age, aBMD, and forearm skinfold thickness on signal quality remains unclear. Understanding these relationships is essential for optimizing CBMT’s clinical utility and interpretation.

In this study, we systematically examined how participant biometric attributes affect CBMT signal quality. First, we developed and validated a fully automated SQI Sequential Gating Scoring System for real-time assessment of frequency response function (FRF) signal quality. We then analyzed the relationship between demographic and biometric factors and signal quality.

## Materials and methods

### Participants

Cortical bone mechanics technology data from 388 postmenopausal women enrolled in a multi-center study investigating the fracture discriminative accuracy of CBMT were used for the current analyses (The Stronger Study; NCT05721898).^32^ The study received approval from the Institutional Review Board, and all participants provided written informed consent. Data collection occurred at four different geographically separated sites between June 2023 and December 2024 using 4 distinct CBMT machines: 1 at Ohio University, 1 at Indiana University Indianapolis, and 2 at the University of Florida (1 in Gainesville and 1 in Jacksonville). Most subjects underwent bilateral testing, though some participants could not be tested on both arms due to recent fractures, rotator cuff issues, or other medical conditions (14 participants did not have their left arm tested and 4 did not have their right arm tested). This resulted in complete data from 758 tests (374 from the left arm and 384 from the right arm).

The inclusion and exclusion criteria have been fully described elsewhere.^32^ In brief, all subjects were post-menopausal females aged 50-80 yr with a BMI between 18.5 and 35 kg/m^2^. Key exclusion criteria included self-reported diseases known to interfere with bone metabolism, residency in a nursing home, active rotator cuff tear/recent shoulder surgery/self-reported severe shoulder pain, terminal illness, and use of systemic glucocorticoids for more than 6 mo in the prior year. One-third of the subjects had experienced a fragility fracture, operationally defined as a self-reported arm or leg fracture caused by a fall from a height of less than 6 inches after the age of 50 yr.

### CBMT testing

Cortical bone mechanics technology conducts a non-invasive, painless, and rapid 3-point bend test on the forearm. For details on the CBMT model used in this study, including photographs and CAD drawings with labeled key components, refer to our prior publication.^32^ This method is grounded in the principles of Euler–Bernoulli beam theory, which describes the behavior of beams subjected to bending forces.^44^ According to this theory, bending a beam induces tensile and compressive stresses, resulting in a bending moment. This concept is extensively applied in material science and engineering to evaluate the mechanical properties of materials under load.^44^

The primary measurement obtained from the CBMT test is flexural rigidity, also known as bending rigidity or flexural stiffness. This mechanical property quantifies the resistance of a structural element (such as the ulna) to bending or flexural deformation. Higher flexural rigidity indicates a greater ability to resist bending, assuming other factors remain constant. Flexural rigidity, denoted as “EI,” is calculated in N·m^2^ by multiplying the Young’s modulus (E), which measures material stiffness or elasticity (in Pascals, N/m^2^), with the second moment of area (I) of the cross-sectional shape (m^4^), a geometric property representing the material distribution around the bending axis.

Cortical bone mechanics technology testing was performed bilaterally (except in the few scenarios noted earlier) and as previously described.^32^ The current protocol takes about 12 min/arm for setup and testing. Prior to testing, the ulna length (L) was measured using electronic calipers (Model 10274876 IP54 electronic calipers with 0.01 mm resolution, MSC Industrial Direct Co.) and the mid-point was marked with indelible ink.

During testing, participants laid supine on the table of the CBMT instrument with care taken to ensure accurate positioning. Essential elements for positioning included (1) ensuring the subject’s styloid process of the distal radius was securely resting on a rigid wrist platform, with the thumb directed towards the ground and the fifth metacarpal facing upwards to maintain a neutral wrist rotation and (2) adjusting the table (horizontally, in the x- [left-right] and y- [head-toe] directions) and the elbow stabilizer to position the elbow and shoulder at 90°, ensuring the humerus is perpendicular and the ulna is parallel to the long side of the table. The wrist platform was then adjusted vertically (in the z-direction) once the elbow was raised to minimize contact between the scapula and table. This alignment optimized the perpendicular relationship between the probe motion axis and the ulna. Wrist clamps were secured to minimize wrist movement, and the elbow stabilizer was firmly placed under the humeral epicondyles and elevated to isolate the forearm from the body. Prior to starting the test, an additional positioning verification was performed by leveling the ulna at its marked midpoint using a small single-axis bubble level.

**Figure 1:**
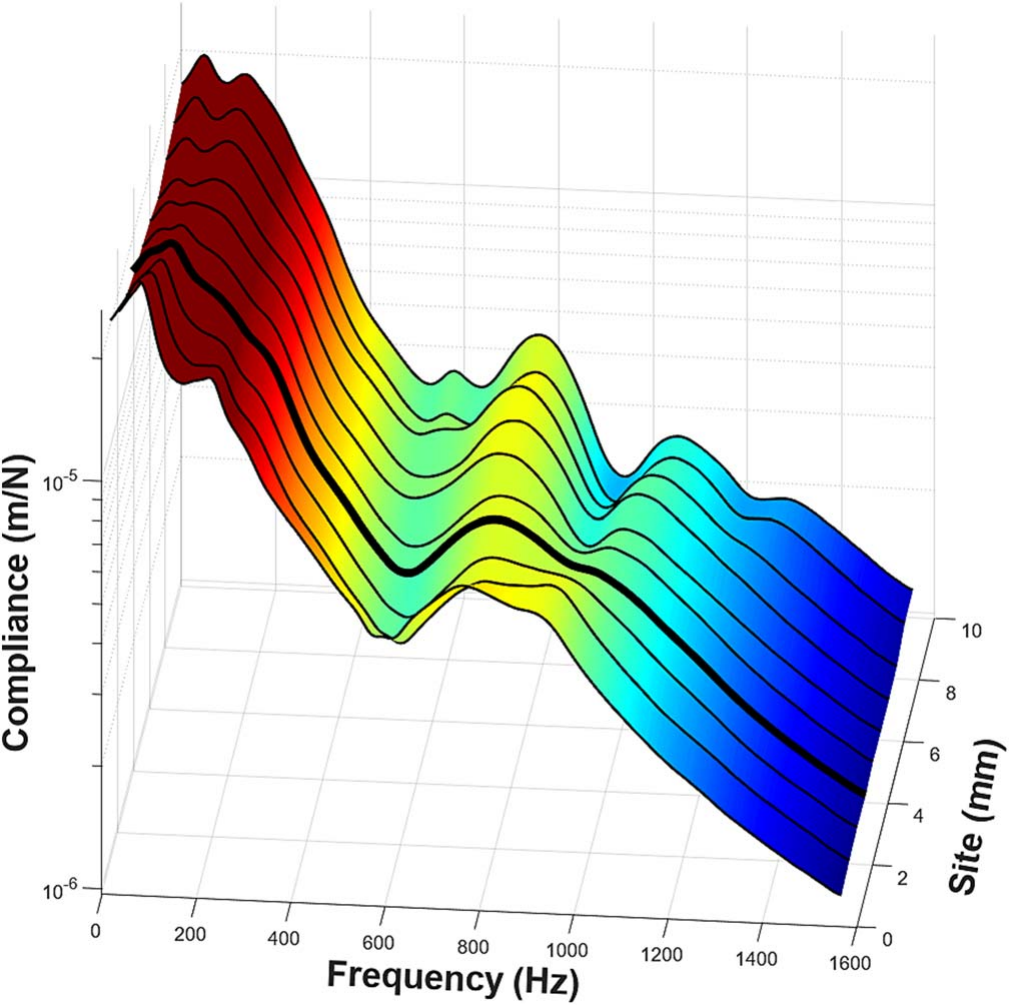
Spatial and spectral variation in frequency response functions (FRFs) across ulnar test sites from a single subject. This 3D surface plot illustrates the compliance (z-axis) across frequency (x-axis) and anatomical site index (y-axis). Data were collected at 10 adjacent sites spaced 1 mm apart, reflecting the CBMT protocol’s spatial sampling strategy. The color gradient depicts the magnitude of compliance, with warmer colors indicating higher values. While there is expected variability across sites, the dominant resonance features, particularly the lower-frequency bone-associated peaks, are consistently present. This visualization highlights how local anatomical and soft tissue variations influence signal fidelity and underscores the importance of sampling across multiple positions to capture high-quality modal signals for analysis. It is important to note that SQI scores evaluate the quality of the experimental FRF signals, not the quality of the model fitting itself. From a translational standpoint, the automated SQI scoring system is intended to guide workflow decisions, such as whether a test should be repeated, and to support consistent signal quality assessment.

For each test, data were collected from 10 discrete sites along the ulna’s diaphysis, spaced at 1 mm intervals to span a total length of 9 mm centered at the midshaft. Each site was evaluated under three distinct vibration amplification conditions. The decision to acquire data from multiple diaphyseal sites is grounded in classical beam theory and the recognition that long bones, like engineered beams, do not exhibit perfectly uniform material or geometric properties along their length.^12^^,^^26^^,^^27^ This spatial sampling strategy was designed to address both anatomical and methodological variability across the bone, including local differences in cortical thickness, cross-sectional geometry, and surrounding soft tissues, all of which can influence signal quality and introduce extraneous vibration modes.^12^^,^^27^ Minor deviations in force probe placement can introduce torsional or off-axis bending modes that distort the FRF (Figure 1), and as a result, some sites are expected to produce poor-quality FRF signals.^12^ Rather than relying on the operator to identify the precise optimal probe location, which is impractical due to anatomical variability, the protocol systematically samples a linear array of sites. Among these, at least one typically approximates ideal antero-posterior bending, producing a clean FRF with distinct modal peaks.^12^ This approach transforms a difficult a priori alignment problem into a solvable post hoc analysis task: identifying and scoring the trials that best reflect the intended mechanical response. In doing so, the strategy increases the likelihood of capturing diagnostically robust FRFs, enhances reliability through spatial averaging, and minimizes the influence of poor-quality signals from anatomically less favorable positions. In total, 22 740 FRF trials were collected (758 tests × 10 sites × 3 amplification levels), serving as the basis for development and validation of the CBMT SQI scoring system. We again note that, given the nature of sampling multiple forearm sites, a substantial portion of these trials is expected to yield lower-quality FRF signals.

The operator initiated the test by positioning the end effector apparatus over the marked midpoint of the ulna. Following this, the automated CBMT instrument directed a Delta robot, which utilizes three linear actuators (Model AINZ9D-B0M0E0, Ultramotion), to lower a force and acceleration probe with a concave ceramic tip into contact with the skin/tissue overlying the ulna bone. The end effector is comprised of a mechanical shaker (Model K2007E01, The Modal Shop, Inc.), an impedance head (Model 288D01, PCB Piezotronics), and a ceramic saddle-shaped patient-contact probe. Once in position, the probe was displaced downward to apply a specified static load (12 N). The shaker was then activated by an excitation signal superimposed on the static load, consisting of a band-limited (20-1600 Hz) chirp sequence that increased and decreased repeatedly (zero mean, 6 N span). The applied force and the resulting acceleration of the ulna-skin complex were measured by the impedance head and sampled at 16 kHz by a 2-channel in-line signal conditioner (Model 485B39, The Modal Shop, Inc.).

**Figure 2:**
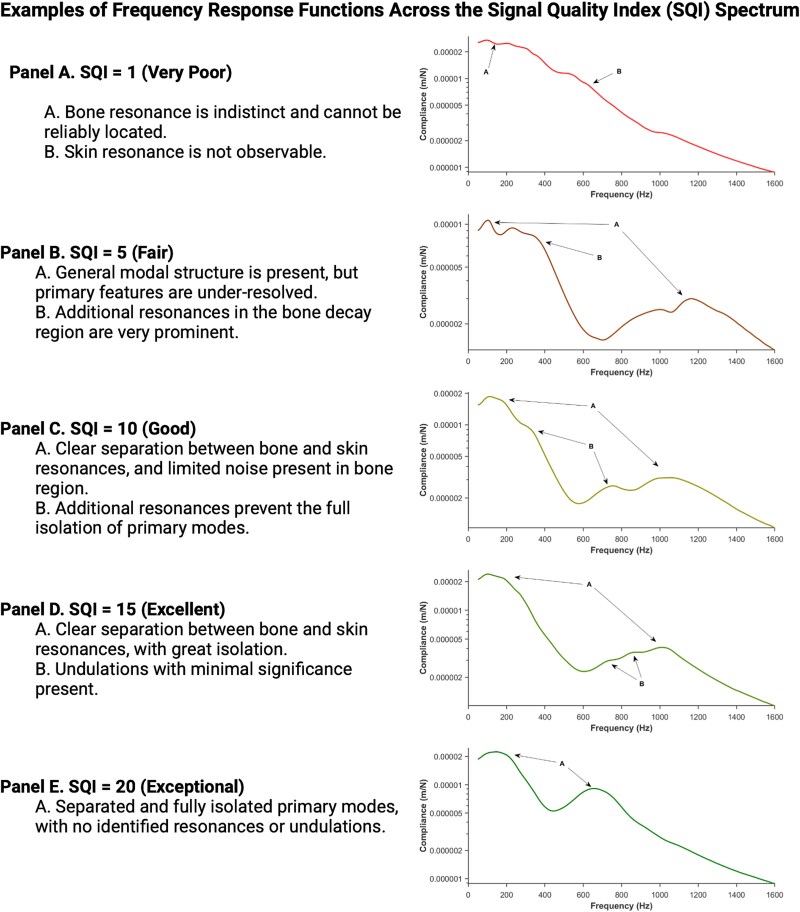
Representative compliance-frequency curves from five different subjects are shown for trials with SQI scores of 1 (very poor), 5 (fair), 10 (good), and 20 (exceptional). These illustrate the progressive improvement in resonance clarity and signal isolation with increasing SQI. At low SQI levels, bone resonance is indistinct and confounded by noise or overlapping features. As SQI increases, modal structure becomes more defined, with clearer separation between bone and skin resonances, reduced noise, and fewer secondary features. Curves are annotated to highlight key characteristics that inform the SQI scoring rubric, including primary mode clarity, modal separation, and noise in the bone decay region. In high-quality FRF signals, the complex compliance FRF signals demonstrate 2 primary resonances. The location and shape of the higher frequency resonance is determined primarily by the mechanical properties of the skin and other soft tissue and the applied static load, while those of the lower frequency resonance are determined primarily by the mechanical properties of the underlying bone.^26^^,^^27^ Both resonances are also affected by damping effects of surrounding soft tissue. Non-biological low frequency (<50 Hz) noise that is attributed to the mechanical system has been filtered and removed. Ulnar flexural rigidity is ultimately quantified based on the compliance FRF model fit. Abbreviations: FRF, frequency response functions; SQI, signal quality indicator.

Proprietary vibration analysis software developed in MATLAB (The MathWorks, Inc.) was used to record the FRF for compliance (displacement divided by force). Displacement was derived from acceleration data through double integration. Figure 2E displays a high-quality complex compliance FRF line, showing resonances around 200 and 800 Hz. The location and shape of the higher frequency resonance are primarily influenced by the mechanical properties of the skin and the applied static load, while the lower frequency resonance is primarily influenced by the mechanical properties of the underlying bone.^26^^,^^27^ Both resonances are also affected by the damping effects of surrounding soft tissue.

EI was calculated using two complementary fitting approaches to improve robustness and reduce sensitivity to outliers in the FRF: (1) global fitting, which applied a two degree-of-freedom, dynamic beam vibration model to the experimentally derived FRF, across the entire frequency range of interest and (2) localized fitting, which utilized a fitted 10th-order polynomial for modal peak extraction and to define subregions of interest where the same 2°-of-freedom model fitting effort was focused. Bone stiffness, K_B_, is the primary outcome of the model fitting and is employed directly in the estimation of EI. Final EI values were calculated as the arithmetic mean of the estimates obtained from both approaches, a strategy supported by cadaveric studies to enhance robustness (unpublished data). EI was computed using the equation EI = K_B_ × L^3^/48, where K_B_ represents the dynamic bending stiffness and L is ulna length. This formulation of EI is derived as the particular solution to the governing differential equation of a simply supported beam, with a pinned support at one end and a roller support at the other, subjected to a central point load. It is a well-known solution commonly presented in classical strength of materials texts.^45^

### Biometric attributes

Descriptive characteristics (eg, age, race, height, weight, arm dominance, etc.) and medical history were collected. Height and weight were objectively measured using calibrated devices.

Dominant and non-dominant hand grip strength was assessed using a hand grip dynamometer (Jamar Plus Digital Dynamometer, Sammons Preston). Following adjustment to hand size, 3 trials were performed with approximately 60 s of rest between trials. If the difference between any two measures on the same hand was >3 kg, an additional trial was conducted. The maximum values for the dominant and non-dominant hands were used in analyses.

Forearm circumference and skinfold thickness were measured on both arms at the mid-point of the ulna, marked with indelible ink for CBMT testing. These measurements were incorporated into the protocol partway through the study and therefore were available only for the final 172 participants. An anthropometric tape measure was used for circumference measurements. Two measurements to the nearest 0.1 cm were taken for each arm and averaged. If the values differed by >0.1 cm, an additional measurement was taken, and the average of the three measurements was used.

For skinfold thickness, the measurement was performed vertically over the mid-point of the ulna using Lange skinfold calipers (Beta Technology). The examiner firmly grasped the subject’s skin between the thumb and index finger, lifting it to include both the skin and subcutaneous fat, but not muscle. The calipers were placed at a 90° angle to the skinfold, approximately 1 cm below the fingers. The examiner slightly released the pressure between the fingers, allowing the calipers to apply greater pressure, and read the needle to the nearest 0.1 mm approximately 4 s after releasing the caliper handle. A minimum of 2 measurements were taken and averaged. If the values varied by >1 mm, an additional measurement was taken, and the average of the 3 measurements was used.

Ulna BMD was measured bilaterally using forearm DXA scans, as previously described. Details of each of the four scanners used can be found in our prior work.^32^ Sites with surgical hardware were excluded. All densitometers underwent cross-calibration using a common Hangartner phantom^46^ to ensure site-to-site comparability, with a linear regression formula applied to standardize scan data from each of the four machines. Standard daily quality assurance tests were conducted, and weekly scans of a Hangartner phantom specific to each study site were performed to document the stability of each DXA machine over time, following International Society of Clinical Densitometry guidelines.^47^ The tolerance limit for stability was set at ±1.5% and all machines remained within this limit throughout the study.

DXA scans were conducted by certified technicians following standardized procedures for participant positioning and scan analysis, adhering to the manufacturer-specific standard methods of operation. DXA operators were trained using a standard operating procedures manual, and all scans underwent centralized quality control assessment at the coordinating center at Ohio University. The coordinating center reviewed all DXA scans to ensure adherence to standardized analysis techniques, providing review comments to the sites and requesting re-analysis or re-scanning as necessary.

### Development of the SQI sequential gating scoring system

**Figure 3:**
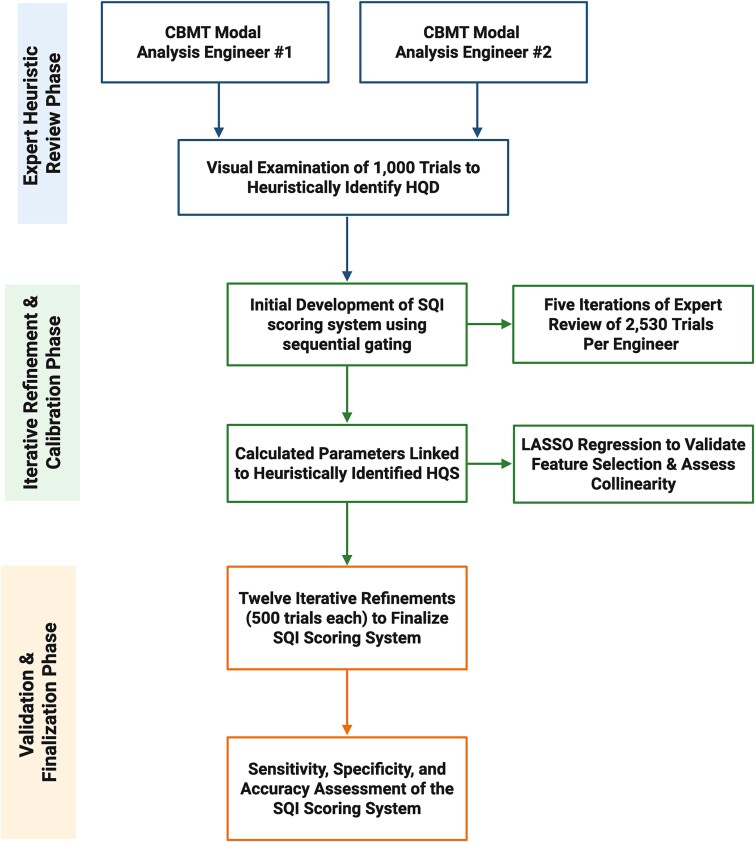
Development and validation workflow for the cortical bone mechanics technology (CBMT) signal quality indicator (SQI) scoring system. This flowchart illustrates the multi-phase process used to develop and validate the SQI scoring system for CBMT signals. The workflow is organized into 3 phases: Expert Heuristic Review, Iterative Refinement and Calibration, and Validation and Finalization. In the first phase (ie, the calibration phase), two experienced modal analysis engineers independently reviewed 1000 CBMT trials to heuristically identify features of high-quality signals (HQS). These insights informed the initial construction of a sequential gating scoring system. In the second phase (ie, the SQI refinement phase), 5 rounds of independent review (2530 trials per engineer) were conducted to refine the system, supported by the calculation of signal-derived parameters and validation through Least absolute shrinkage and selection operator (LASSO) regression to optimize feature selection and reduce collinearity. The final phase involved 12 iterative refinements (500 trials each) to finalize the scoring system, culminating in a quantitative assessment of sensitivity, specificity, and accuracy. Color-coded elements correspond to each development phase. Abbreviations: HQD, high-quality signal; SQI, signal quality indicator.

The development and validation process of the SQI scoring system is summarized in Figure 3, which outlines the multi-phase workflow from expert heuristic review through iterative refinement and final validation.

To initiate development, 2 experienced CBMT modal analysis engineers (A.D. and M.S.) visually examined 1000 randomly selected trials from the full dataset of 22 740 FRF trials. Their goal was to identify the key characteristics that distinguished high-quality from low-quality signals. Features, such as distinct vibration modes, well-defined peak structures, and high signal-to-noise ratios, were heuristically identified as markers of high-quality signal behavior. Based on these insights, the engineers constructed an initial sequential gating system that assigned a SQI score from 1 to 20, with higher scores indicating better signal quality. This tiered structure functioned such that signals had to pass through a series of rules (gates), with only those meeting all predefined criteria receiving high SQI scores.

To provide methodological transparency, we detail here the iterative refinement process used to finalize the SQI scoring rubric. Outcomes are reported separately in the Results section.

The system underwent 17 total SQI refinement iterations, each involving a new, independent set of approximately 500 FRF trials. Prior to these SQI refinement iterations, a series of calibration rounds (*n* = 5) was conducted to align operator scoring and ensure consistent interpretation of signal quality. During these calibration rounds, engineers independently rated trial quality without access to one another’s evaluations or a predefined rubric, allowing for the development of a shared intuitive framework. By the end of this calibration phase, inter-rater reliability was exceptionally high (intraclass correlation coefficient = 0.985), and Bland–Altman analysis revealed minimal systematic bias (−0.075, 95% CI, −0.045 to −0.105). Formal refinement of the SQI gates began only after this calibration phase, ensuring that scoring consistency and the operational definition of quality were co-developed in parallel.

After the fifth SQI refinement iteration, we extracted multiple signal-derived features from the FRF signal using MATLAB. These candidate predictors included peak count, resonance alignment, mode separation, harmonic clarity, noise metrics, and artifact detection. We then applied LASSO regression^48^ as a single diagnostic step to evaluate the relative importance of these features and assess multicollinearity. In this model, the dependent variable was the expert-assigned SQI score for each trial. LASSO, a regularization technique that imposes an L1 penalty on regression coefficients,^49^ was not used to build a final predictive model but rather to guide refinement of the feature set. The model was trained on 18 192 trials (80% of the sample) and validated on 4548 trials (20%). Using an optimal regularization parameter (λ = 0.12), the model achieved an adjusted *R*^2^ of 75.5% in the training set and 68.3% in the test set. These results underscored the predictive value and generalizability of the selected features. This analysis facilitated the identification and removal of weak or redundant predictors, enabling a more parsimonious and interpretable scoring system. The final refined feature set was codified into a formal rule-based system, replacing subjective evaluation in all subsequent iterations (Iterations 6-17).

The rule-based system was tested and adjusted over 12 additional iterations (Iterations 6-17). In each cycle, analysts assessed whether rule-generated SQI scores matched their qualitative expectations, flagging instances where scores were too high or too low. Although the same experts were used across iterations, introducing a potential training effect, this approach was deliberate, as their evaluations served as the gold standard for calibration.

**Figure 4:**
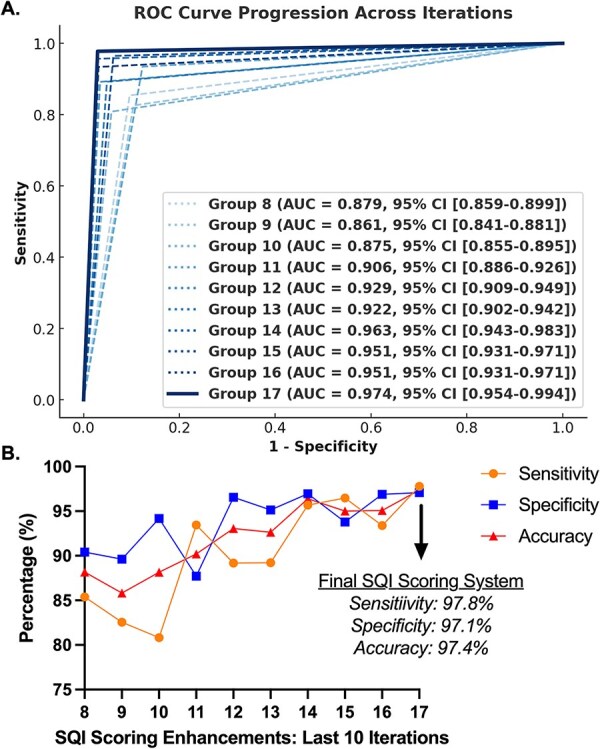
Demonstration of construct validity of the signal index (SQI) sequential grading scoring system. (A) Receiver operating characteristic (ROC) area under the curve (AUC) values plotted across ten iterative refinements of the SQI scoring system (iterations 8-17). Each point reflects the system’s AUC based on comparison to expert-derived reference labels on an independent validation set. (B) Sensitivity, specificity, and accuracy values across the same 10 iterations. Note that all three metrics were calculated independently for each iteration. These plots collectively demonstrate performance stabilization and help identify the optimal iteration. Abbreviations: SQI, signal quality indicator.

Each iteration yielded performance metrics based on agreement with expert assessment. Figure 4A shows area under the ROC curve (AUC) values across the final ten iterations (Iterations 8-17), while Figure 4B displays corresponding sensitivity, specificity, and accuracy. Although performance metrics began to plateau after Iteration 14, refinement continued through Iteration 17 to confirm that the scoring system was stable, generalizable, and not overfitting transient features in the training data. The final system achieved excellent performance (AUC = 0.974; sensitivity = 97.8%; specificity = 97.1%; accuracy = 97.4%).

Each rule within the system acts as a gating mechanism, progressively filtering trials based on increasingly specific signal quality criteria. Early rules address fundamental signal integrity (eg, presence of valid peaks), while later rules evaluate noise characteristics, modal separation, and artifact count. This architecture ensures that only signals with stable, interpretable mechanical signatures are retained for clinical or research interpretation. A summary of the scoring framework is presented in Table 1, with technical scoring logic outlined in Table 2.

**Table 1:** Summary of signal quality indicator (SQI) score criteria for CBMT signal characterization. This table outlines the defining features associated with each SQI score range based on modal behavior observed in CBMT frequency response functions. Full scoring logic is detailed in Table 2.

SQI score	Descriptor	Interpretation
**1-2**	Very Poor	Very poor signal characteristics; modal separation is absent, with overlapping bone, skin, and low-frequency noise peaks, and no identifiable antiresonances or inter-peak spacing to support reliable mode discrimination.
**3-4**	Poor	Poor signal characteristics; modal separation is limited, with bone and skin resonances present but exhibiting low prominence, hindering reliable mode discrimination.
**5-6**	Fair	Fair signal characteristics; partially defined modal separation with an atypical decay profile following the bone peak, indicating deviation from expected model characteristics.
**7-8**	Moderate	Moderate signal characteristics; modal features are present but occur outside expected physiological frequency ranges, reflecting deviations from typical bone and soft tissue response profiles.
**9-10**	Good	Good signal characteristics; defined modal structure with reduced noise supports proper mode separation, and additional resonances are present but do not diminish the prominence of the primary peaks.
**11-12**	Very Good	Very good signal characteristics; prominent modal separation with clear primary peaks and no more than two additional resonances outside the primary modes.
**13-14**	Great	Great signal characteristics; strong modal separation with well-isolated primary peaks and no more than one additional resonance outside the primary modes.
**15-16**	Excellent	Excellent signal characteristics; well-resolved modes with a stable signal-to-noise ratio and no more than four low-prominence undulations.
**17-18**	Outstanding	Outstanding signal characteristics; highly consistent modal behavior with near-complete clarity and no more than two low-prominence undulations.
**19-20**	Exceptional	Exceptional signal characteristics; benchmark-level modal definition with complete clarity and no deviations across evaluated criteria.

Abbreviation: CBMT, cortical bone mechanics technology

**Table 2:** Computational feature definitions and scoring criteria for the CBMT signal quality indicator (SQI). This table details the rule-based feature checks used in the sequential gating system for SQI assignment. Proprietary thresholds (THRESH) are excluded for intellectual property protection.

**1) Overall Signal Quality: Score Range 1-2**	
**a) Coefficient of determination (*R*^2^) exceeds THRESH due to uniform decay across the frequency range, indicating an absence of localized resonance behavior attributable to bone or skin modes.**	**Score 1**
**b) Total prominence percentage—defined as the ratio of the combined prominence of identified bone and skin peaks to the sum of all peak prominences—falls below THRESH, indicating that diagnostically relevant modal content is overwhelmed by non-physiological or spurious peaks.**	**Score 1**
**c) The magnitude of the identified bone peak is lower than those present in the low-frequency noise region (<THRESH), impairing the model’s ability to represent bone-related dynamics.**	**Score 2**
**2) Regional Mode Prominence: Score Range 3-4 (Low Threshold Criteria)**	
**a) The bone resonance prominence percentage—defined as the prominence of the bone resonance divided by the sum of all prominences preceding the antiresonance—falls below the low-threshold criterion, indicating partial emergence of the bone mode with insufficient separation from adjacent features.**	**Score 3**
**b) The skin resonance prominence percentage—defined as the prominence of the skin resonance divided by the sum of all prominences following the antiresonance—falls below the low-threshold criterion, reflecting a non-discernable skin mode with overlap from surrounding features.**	**Score 4**
**3) Bone Resonance and Decay Range Validation: Score Range 5-6**	
**a) A resonance appears within the post-bone decay region (THRESH-THRESH), inconsistent with the expected response profile, indicating the presence of an erroneous feature.**	Score 5
**b) The half-width of the identified bone resonance falls below THRESH, indicating a damping profile inconsistent with that typically observed for bone modes.**	**Score 5**
**c) A low-prominence undulation is detected in the post-bone decay region with a prominence exceeding THRESH and a slope below THRESH, suggesting a feature that may significantly interfere with model fit.**	**Score 6**
**4) Modal Feature Range Verification: Score Range 7-8**	
**a) The frequency of the bone resonance falls outside the expected physiological range (THRESH-THRESH), representing a deviation from typical anatomical response characteristics.**	**Score 7**
**b) The selected antiresonance frequency lies outside the expected physiological range (THRESH–THRESH), reducing alignment with standard mode structure and limiting confidence in model fidelity.**	**Score 8**
**5) Regional Mode Prominence: Score Range 9-10 (High Threshold Criteria)**	
**a) The bone resonance prominence percentage—defined as the resonance prominence divided by the sum of all prominences preceding the antiresonance—falls below the high-threshold criterion, indicating a discernible bone mode with moderately limited separation from adjacent features.**	**Score 9**
**b) The skin resonance prominence percentage—defined as the resonance prominence divided by the sum of all prominences following the antiresonance—falls below the high-threshold criterion, reflecting a visible skin mode with modest overlap from surrounding features.**	**Score 10**
**6) Total Resonant Feature Count: Score Range 11-12**	
**a) The total number of identified resonant features exceeds four, and more than five low-prominence undulations are present, indicating a well-defined primary modal structure with modest contributions from secondary features that mildly reduce modal isolation.**	**Score 11**
**b) The total number of full resonant features exceeds four, reflecting a clearly interpretable modal landscape with mild influence from additional resonances.**	**Score 12**
**7) Total Resonant Feature Characteristics: Score 13-14**	
**a) The total number of resonant features exceeds three, indicating a clean and coherent modal structure with a single additional resonance outside the primary modes that does not substantially affect interpretability.**	**Score 13**
**b) The total prominence percentage falls just below THRESH, suggesting that diagnostically relevant modes are clearly defined, though their relative dominance is slightly reduced by secondary modal content.**	**Score 14**
**8) Undulation Profile Evaluation: Score 15-16**	
**a) The total number of undulations across the frequency range exceeds four, indicating a strong primary modal structure with minor non-modal features that minimally reduce overall signal clarity.**	**Score 15**
**b) One or more undulations in the post-bone resonance decay region exceed the prominence threshold, suggesting limited persistence of low amplitude features outside of the primary bone resonance, which may minimally impact model resolution.**	**Score 16**
**9) Final Undulation Assessment: Score Range 17-18**	
**a) The number of undulations is greater than two, reflecting a highly stable frequency response with the presence of trace non-primary modal content.**	**Score 17**
**b) The bone resonance prominence percentage falls below a threshold of 1.0, confirming that the bone mode accounts for nearly all pre-antiresonance signal energy, with negligible contribution from secondary features.**	**Score 18**
**10) Benchmark Signal Quality? Score Range 19-20**	
**a) The skin resonance prominence percentage falls below a threshold of 1.0, indicating that the skin mode accounts for nearly all post-antiresonance signal energy, with only inconsequential contribution from secondary features.**	**Score 19**
**b) All evaluative criteria are fully satisfied with no deviations; the signal exhibits ideal modal definition, clarity, and stability, qualifying as a benchmark-level response.**	**Score 20**

Abbreviation: CBMT, cortical bone mechanics technology.

To visually demonstrate the spectrum of FRF signal quality captured by the SQI, a multi-panel figure (Figure 2A-E) presents representative examples across the SQI range. These examples illustrate how differences in peak clarity, modal separation, and noise profiles correspond to the scoring criteria embedded within the system.

Importantly, the final SQI scoring system is fully automated and embedded within the CBMT software platform. Following each vibration trial, the SQI score is calculated and displayed within seconds, enabling real-time feedback on signal quality. This eliminates the need for post hoc human review and ensures consistent application across clinical and research environments. The automated implementation enhances translational feasibility by supporting high-throughput testing, scalability, and clinical decision-making.

### Statistical analyses

#### Validation of the SQI sequential gating scoring system

To evaluate content validity, we evaluated the SQI scoring system’s classification performance using receiver operating characteristic (ROC) analysis, using expert assessment as the gold standard. Agreement was defined as an automated SQI score falling within ±1 point of the expert-assigned value; scores outside this range were considered to be misclassified. This evaluation was conducted across the final 10 refinement iterations. Receiver operating characteristic curves were generated to quantify the system’s ability to successfully replicate expert-level scoring, with corresponding sensitivity, specificity, and accuracy values reported.

#### Impact of biometric attributes on CBMT signal quality

Due to observed differences in SQI scores between limbs (see “Results” section), separate analyses were conducted based on limb dominance. Paired *t*-tests were used to compare SQI scores between the dominant and non-dominant sides. We also performed separate analyses using: (1) the highest SQI score observed across the 10 anatomical test sites on each arm (“single best SQI score”) and (2) the average of the three highest SQI scores across these sites. The relationships between SQI scores and selected biometric attributes were examined using Spearman rank correlation coefficients (ρ), appropriate for the ordinal nature of SQI scores ranging from 1 to 20. We also performed a one-way ANOVA followed by a Bonferroni post-hoc test to compare SQI scores among participants with BMI categories of <25.0 (normal BMI group), 25.0-29.9 (overweight BMI group), and ≥30.0 (obese BMI group) kg/m^2^. To examine whether CBMT signal quality varied across different levels of ulnar EI, participants were stratified into three EI groups based on the distribution of EI values separately for each limb. Specifically, thresholds were defined using ±1 SD from the mean EI value. For the dominant limb, participants with EI < 22.2 N·m^2^ were categorized as low EI, those with EI between 22.2 and 50.4 N·m^2^ as mid EI, and those with EI > 50.4 N·m^2^ as high EI. Corresponding thresholds for the non-dominant limb were <21.3, 21.3-45.9, and >45.9 N·m^2^, respectively. Signal quality indicator scores were then compared across these EI strata using one-way ANOVA with Bonferroni-corrected post hoc tests. Mean and SD were used for descriptive statistics. All statistical analyses were performed using SPSS, with a pre-set alpha level of significance of 0.05.

## Results

### Content validity of the SQI sequential gating scoring system

The final iteration of the SQI scoring system demonstrated excellent agreement with expert assessments, with an AUC of 0.974 (95% CI: 0.954-0.994), sensitivity of 97.8%, specificity of 97.1%, and overall accuracy of 97.4%. Figure 4A displays the progression of AUC values across the final ten iterations (Iterations 8-17), while Figure 4B shows corresponding sensitivity, specificity, and accuracy metrics. These results highlight the system’s refinement and stabilization over time, culminating in a robust and reliable automated scoring tool.

### Study participants

Participants had an average age of 66.7 ± 6.2 yr. Their height, weight, and BMI were 1.63 ± 0.06 m, 68.9 ± 11.9 kg, and 25.9 ± 3.9 kg/m^2^, respectively. Dominant and non-dominant hand grip strength were 24.8 ± 6.6 and 23.2 ± 6.4 kg, respectively. aBMD values for the one-third distance and total ulna in the dominant arm were 0.629 ± 0.094 and 0.473 ± 0.078 g/cm^2^, respectively. aBMD values for the one-third distance and total ulna in the non-dominant arm were 0.628 ± 0.087 and 0.473 ± 0.070 g/cm^2^, respectively.

### SQI scores and limb dominance

The single best SQI score did not significantly differ between limbs (Dominant side SQI: 12.2 ± 4.7 vs non-dominant side SQI: 12.8 ± 4.5; *p* = .091). However, when comparing the average of the three best SQI scores, the dominant side exhibited a slightly higher SQI score (11.5 ± 4.5) compared to the non-dominant side (10.7 ± 4.6) (*p* = .014).

### Relationship between SQI scores and biometric attributes

**Figure 5:**
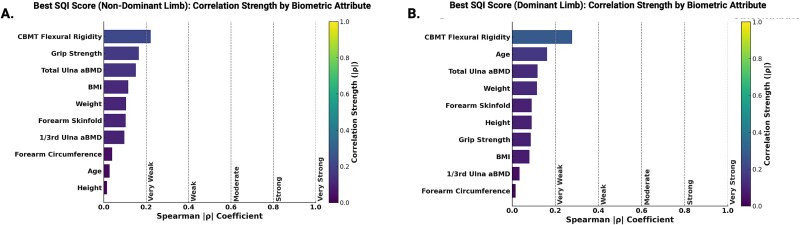
Correlation strength between biometric attributes and CBMT-derived signal quality indicator (SQI) scores for the best site in each limb. Spearman rank correlation coefficients (|ρ|) are shown for 10 biometric and physiological variables relative to the best (ie, highest scoring) SQI value from the non-dominant limb (A) and the dominant limb (B)**.** All correlations were weak or very weak (|ρ| < 0.3), indicating minimal association between biometric variation and SQI performance. Vertical dashed lines denote conventional thresholds for correlation strength: very weak (|ρ| = 0.0-0.19), weak (0.20-0.39), moderate (0.40-0.59), strong (0.60-0.79), and very strong (0.80-1.0). Color intensity reflects correlation magnitude using a consistent heatmap scale across both panels. Abbreviations: CBMT, cortical bone mechanics technology.

There were few statistically significant associations between SQI scores and biometric attributes (Table 3; Figure 5), with all observed significant correlations being weak (ρ < 0.20).

**Table 3:** Spearman rank correlation coefficients between CBMT signal quality index (SQI) scores and selected biometric attributes.

	Best SQI score		Average SQI score	
	Non-dominant	Dominant	Non-dominant	Dominant
**Age**	0.027	0.165^**a**^	0.067	0.162^**a**^
**Height**	−0.015	−0.062	−0.014	−0.090
**Weight**	−0.105^**a**^	−0.105^**a**^	−0.068	−0.115^**a**^
**BMI**	−0.115^**a**^	−0.079	−0.082	−0.080
**Forearm circumference**	−0.039	0.004	−0.075	−0.016
**Forearm skinfold**	−0.103	−0.097	−0.043	−0.090
**1/3 ulna aBMD**	−0.097	−0.096	−0.058	−0.034
**Total ulna aBMD**	−0.151^**a**^	−0.166^**a**^	−0.099	−0.118^**a**^
**CBMT flexural rigidity**	−0.221^**a**^	−0.251^**a**^	−0.261^**a**^	−0.278^**a**^
**Grip strength**	0.165^**a**^	0.104	0.110^**a**^	0.086

(1) The “Best Signal Quality Index (SQI) Score” reflects the top score observed among 30 trials (conducted at 10 sites with three different amplitudes). Whereas the “Average SQI Score” represents the mean of the top three trials from the total of 30 trials conducted.

a (2) indicates *p*-value ≤.05.

(3) Forearm circumference and skinfold thickness measurements were conducted only for the final 172 subjects enrolled in the study.

Abbreviations: aBMD, areal BMD; CBMT, cortical bone mechanics technology.

There were very weak negative associations between SQI scores and body weight for both the best SQI score (non-dominant: ρ = −0.105, *p* = .049; dominant: ρ = −0.105, *p* = .047) and the average SQI score on the dominant side (ρ = −0.115, *p* = .031), indicating that higher body weight was slightly associated with lower signal quality. Similarly, a very weak negative correlation was found between the best SQI score and BMI for the non-dominant side (ρ = −0.115, *p* = .031).

There were weak negative associations between SQI scores and total ulnar aBMD for both the best SQI score (non-dominant: ρ = −0.151, *p* = .005; dominant: ρ = −0.166, *p* = .002) and average SQI score on the dominant side (ρ = −0.118, *p* = .028).

There were also weak negative correlations between SQI scores and CBMT-derived flexural rigidity. This was consistent across both limbs and both scoring approaches (ρ = −0.221 to −0.278, all *p* < .01), indicating that higher bending stiffness was associated with slightly lower signal quality.

There was a very weak positive correlation between SQI scores and age for the dominant side (Best SQI: ρ = 0.165, *p* = .002; Average SQI: ρ = 0.162, *p* = .002), suggesting that older participants had slightly better signal quality. Additionally, a weak positive association was found between the best SQI score and grip strength for the non-dominant side (ρ = 0.165, *p* = .002), with a similar association observed for the average SQI score (Non-Dominant: ρ = 0.110, *p* = .040).

There was no significant correlation between SQI scores and height for either the best or average SQI scores (Table 3; Figure 5). Similarly, there were no significant correlations between SQI scores and forearm circumference or skinfold measurements or between SQI scores and the one-third ulna aBMD (Table 3; Figure 5).

### Groupwise comparisons of SQI by BMI and EI

**Figure 6:**
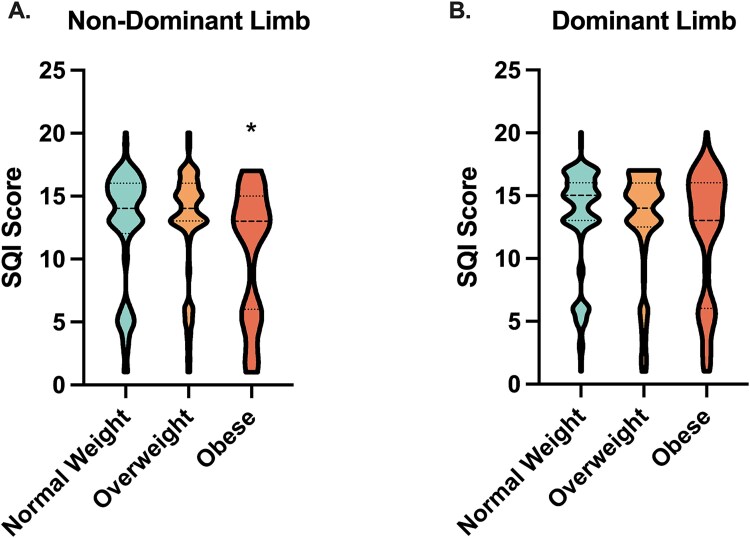
CBMT signal quality indicator (SQI) subgroup comparisons. Violin plots of the best SQI scores by BMI category (normal: <25 kg/m^2^, overweight: 25-29.9 kg/m^2^, obese: ≥30 kg/m^2^) for the non-dominant (A) and dominant (B) limbs. The asterisk indicates significantly lower SQI score in the obese group for the non-dominant limb, with no difference between the normal and overweight groups. No differences were observed for the dominant limb. Violin plots showing SQI scores for the non-dominant (A) and dominant (B) limbs across BMI categories. Distributions are truncated, with wider sections indicating greater data density. Horizontal lines represent medians. Abbreviations: CBMT, cortical bone mechanics technology.

A one-way ANOVA revealed a significant main effect of BMI category on SQI scores for the non-dominant limb (Best SQI: *p* = .004; Average SQI: *p* < .001), but not for the dominant limb (Best SQI: *p* = .079; Average SQI: *p* = .130). Post-hoc analyses for the non-dominant limb showed no significant difference in best SQI scores between the normal and overweight BMI groups (12.52 ± 4.62 vs 12.84 ± 4.29; *p* = 1.0). However, individuals in the obese BMI group had a significantly lower best SQI score (10.54 ± 5.15), approximately 2 points lower than the other two groups (*p* < .05) (Figure 6). A similar pattern was observed for the average SQI score: the normal and overweight BMI groups did not differ (10.88 ± 4.46 vs 11.47 ± 4.21; *p* = .80), while the obese group had a significantly lower score (8.86 ± 4.99; *p* < .05). Thus, while we did observe an effect of a higher BMI on CBMT signal quality, even among obese subjects the SQI was, on average, still in an acceptable range, supporting the general robustness of CBMT signal acquisition across a wide range of BMI.

A one-way ANOVA revealed a significant main effect of EI category on SQI scores for both the non-dominant limb (Best SQI: *p* < .001; Average SQI: *p* < .001) and the dominant limb (Best SQI: *p* = .02; Average SQI: *p* = .02). Post-hoc analyses for the non-dominant limb showed that individuals in the high EI group had significantly lower best SQI scores (9.50 ± 5.71) compared to those in the low (13.91 ± 4.19; *p* < .001) EI groups. The difference between the low and mid (12.49 ± 4.25) EI groups did not reach statistical significance (*p* = .10). A similar pattern was observed for the average SQI score: the high EI group had lower scores (8.17 ± 5.15) than both the mid (10.79 ± 4.24; *p* < .01) and low (12.86 ± 4.26; *p* < .001) EI groups. For the dominant limb, post-hoc comparisons showed that best SQI scores were modestly lower in the high EI group (11.82 ± 5.23) compared to the low EI group (14.51 ± 3.59; *p* = .02), though no significant difference was observed between the low and mid (12.68 ± 4.39) (*p* = .06) or the high and mid EI groups (*p* = .63). For average SQI scores, the high EI group (10.57 ± 4.99) again differed significantly from the low EI group (13.23 ± 3.85; *p* = .02), with no difference between the low and mid (11.38 ± 4.41) (*p* = .06) or the high and mid groups (*p* = .69). These differences in SQI scores across EI categories were statistically significant but modest in magnitude (~2 to 3 points) with the high EI groups still maintaining SQI scores generally in the “good” range, supporting the general robustness of CBMT signal acquisition across a wide range of bone strengths.

## Discussion

The primary objective of this study was to evaluate the robustness of CBMT signal characteristics across a broad range of participant biometric profiles. To address this aim, we developed and validated a Sequential Gated SQI Scoring System, which demonstrated strong construct validity. We then examined how several biometric factors—including age, body weight, BMI, forearm aBMD, grip strength, and soft tissue thickness—related to SQI scores. Although a few associations emerged, all observed correlations were weak (ρ < 0.20), indicating that these biometric attributes exert only minimal influence on CBMT signal characteristics. Participants with higher BMI and EI values exhibited slightly lower SQI scores on average, but this difference was modest (~2 points, see Table 1) and unlikely to meaningfully affect signal interpretation.

One possible contributor to the subtle reduction in SQI scores among individuals with obesity is the dampening effect of excess soft tissue on vibrational energy. Increased soft tissue mass may alter transmission dynamics and attenuate resonant frequencies, thereby reducing the fidelity of the FRF. In the present study, a uniform static preload was applied across all participants, regardless of BMI. Anecdotally, we have observed that applying a higher static preload in individuals with greater soft tissue mass often improves signal quality. From a theoretical standpoint, this observation is consistent with known mechanical principles previously discussed in prior MRTA development work: a greater preload may displace interstitial fluid and compress the soft tissue, reducing its thickness and increasing its effective stiffness.^50^^,^^51^ Conceptually, these changes would shift soft tissue resonances into higher frequency ranges and enhance mechanical coupling between the probe and underlying bone, ultimately improving the signal-to-noise ratio. Although this hypothesis was not formally tested in The STRONGER Study, it represents a compelling avenue for future research, particularly in populations with higher adiposity.

Another notable finding was the weak but statistically significant negative association between CBMT-derived flexural rigidity and SQI scores. Although this may initially appear counterintuitive, it is likely attributable to subtle changes in the vibrational behavior of stiffer bones. Specifically, bones with higher EI values may exhibit sharper or higher-frequency resonance peaks, which can generate additional resonant responses, either due to end-condition effects or interactions between the ulna and radius through the interosseous membrane. These additional resonances can increase noise near the primary modal features, potentially reducing the SQI by interfering with the predefined signal quality thresholds. Conversely, in bones with low EI values, where resonance peaks are dull or heavily damped, it becomes difficult to distinguish the bone and soft tissue vibrational modes. This is likely due to reduced vibrational energy transmission through the surrounding soft tissue.

A known factor influencing signal fidelity is the static preload applied between the measurement probe and the ulna. In this study, the preload was fixed at 12 N, an empirically determined value based on prior (unpublished) cadaveric testing deemed suitable for this cohort. Nonetheless, natural variability resulted in some outliers along the Gaussian distribution, contributing to expected signal noise. In the future, it is possible that the preload could be tuned on a subject-specific basis to improve signal quality. Despite the consistency of this association, its effect size was small and is unlikely to meaningfully impact the clinical utility of CBMT.

The weak associations between biometric characteristics and SQI scores suggest that CBMT can be consistently applied across a wide range of body types and clinical presentations. This broad applicability is critical for ensuring that individuals with diverse anthropometric and skeletal profiles—such as those with obesity or low bone mass—can benefit from CBMT’s diagnostic and monitoring potential. Moreover, the ability to generate high-quality signal across varied biometric conditions conceptually enhances the reliability, scalability, and overall clinical utility of CBMT in real-world settings.

There is a critical need to improve the diagnosis of osteoporosis, as better identifying individuals at risk for fractures would allow for more targeted preventive measures against fragility fractures. The effectiveness of DXA-derived aBMD in assessing bone health and treatment responses is significantly limited.^5–15^^,^^17–19^^,^^22–24^ One limitation stems from aBMD’s inability to fully capture bone strength, addressing only one aspect of it.^5^^,^^10^^,^^23^^,^^24^ Since bone strength is crucial in preventing fractures,^52^^,^^53^ there has been a longstanding demand for the development of new methods to assess bone strength more comprehensively.^10^^,^^21^^,^^22^

CBMT noninvasively quantifies flexural rigidity (EI), a biomechanical property that governs how bone resists deformation under bending loads. While it does not induce failure, CBMT-derived rigidity has been shown to strongly correlate with whole-bone failure load under 3-point bending conditions, making it a powerful surrogate for assessing mechanical bone strength.^12^^,^^26^^,^^27^ Using a dynamic 3-point bending test, CBMT’s measurements reflect the cumulative effects of all factors influencing the ulna at the whole bone level. This includes geometry, tissue material properties and composition, porosity, microarchitecture, nanoscale collagen cross-linking, and protein-mineral bonding.^21^ Therefore, CBMT effectively captures the combined influences of bone quality and quantity on the ulna’s load-bearing capacity.

While this study provides valuable insights into the robustness of CBMT signal quality, it has some limitations. The study population consisted exclusively of postmenopausal women aged 50-80 yr with a BMI range of 18.5-35 kg/m^2^, which may limit the generalizability of the findings to men and other demographic groups. Future research is needed to determine how CBMT signal quality varies across different populations, including men and younger individuals. Additionally, while this study focused on several key biometric attributes, it did not assess the impact of these attributes on test reproducibility. Although signal quality may not be significantly affected by factors such as body mass, subject positioning and test reproducibility could be influenced. Additionally, the study did not examine other potential contributors to variability, such as body composition and anatomical differences like ulnar bowing. Future studies should explore these factors to provide a more comprehensive understanding of both CBMT signal quality and test reproducibility.

A key strength of the SQI system is its full automation. The scoring algorithm is embedded within the CBMT software, allowing real-time evaluation of each FRF trial immediately upon acquisition. This implementation eliminates the need for post hoc review, ensures consistency across users and sites, and facilitates high-throughput testing. From a translational perspective, automated signal quality scoring is essential for integration into clinical workflows (eg, should a test be repeated), and the SQI system presented here fulfills that requirement.

**Figure 7:**
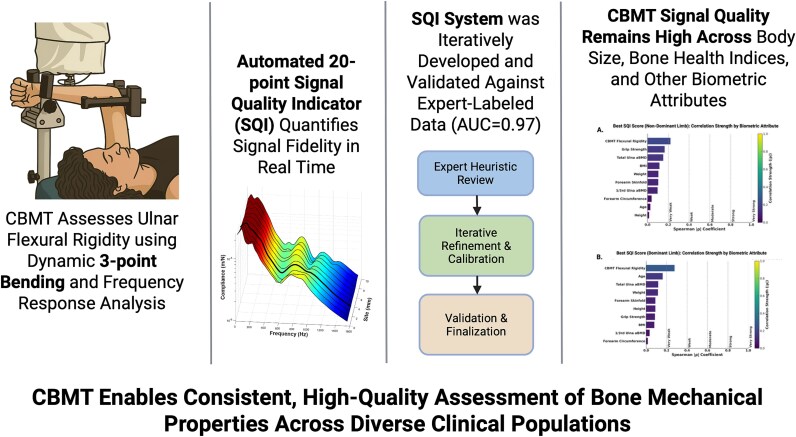
Graphical abstract summarizing the CBMT signal quality indicator (SQI) system, from device function to validation and biometric performance**.** CBMT non-invasively measures ulnar flexural rigidity using dynamic 3-point bending combined with vibration-based analysis. A robotic system moves the loading point along the mid-forearm to 10 discrete sites. A fully automated 20-point SQI algorithm quantifies signal fidelity in real time, developed through expert-guided calibration and achieving high classification accuracy (AUC = 0.97). The system demonstrates robust performance across a wide range of biometric profiles, with SQI scores showing minimal correlation with 10 anthropometric and physiological variables, including BMI. Abbreviations: AUC, area under the curve; CBMT, cortical bone mechanics technology.

In conclusion, this study developed and validated a CBMT SQI scoring system by demonstrating that it reliably reproduces expert evaluations of FRF signal quality, with high classification accuracy in an independent validation set. Among postmenopausal women, the quality of CBMT FRF signals, as characterized by descriptive categories within the validated SQI scoring system, was consistently within the moderate, good, or excellent range across a wide span of biometric profiles. While minor associations were observed—most notably a modest reduction in signal quality among those with obesity—these associations were very weak to weak and unlikely to impact clinical utility. Overall, the findings support the feasibility of using CBMT for bone health assessment by demonstrating that high-quality mechanical signals can be consistently acquired across a wide range of biometric profiles. Given that CBMT directly quantifies flexural rigidity, a key determinant of whole bone strength, this approach may provide a complementary and biomechanically grounded measure of skeletal health alongside traditional diagnostics. The graphical abstract (Figure 7) summarizes the overall study findings and highlights the robustness of the SQI system across biometric variability.

## Data Availability

The data that support the findings of this study are available from the corresponding author upon reasonable request and subject to approval. The available data comprise reduced datasets derived from the original research data.
